# A Giant Cell Fibroma and Focal Fibrous Hyperplasia in a Young Child: A Case Report

**DOI:** 10.1155/2012/370242

**Published:** 2012-06-03

**Authors:** Rodney J. Vergotine

**Affiliations:** Department of Pediatric Dentistry, University of Illinois at Chicago, Chicago, IL 60607, USA

## Abstract

A case of two fibrotic lesions of the oral mucosa in a 17-month-old African-American female is reported. Both lesions occurred on the anterior maxilla, one lesion pedunculated on the buccal attached gingiva and the other lesion sessile on the palate. Histological examination characterized the buccal lesion as focal fibrous hyperplasia (FFH) and the palatal lesion as a giant cell fibroma (GCF). A case is made for continuing the consideration of GCF as a histologically distinct entity from FFH but that no difference in clinical impact between the two lesions exists.

## 1. Introduction

Focal fibrous hyperplasia or fibroma is considered the most common benign soft tissue growth in the oral cavity [1, 2]. This lesion has a predilection for females, occurs in patients older than 30 years, is a few centimeters in diameter, pedunculated or sessile, and occurs frequently on the gingiva or buccal mucosa. Chronic irritation or trauma is frequently identified as the causative factor. Treatment of the fibroma involves surgical excision, and recurrences are very infrequent. In 1974 Weathers and Callihan identified a distinct entity within previously identified fibroma lesions. This was called the giant cell fibroma, a lesion that is specifically distinguished by the presence of stellate/giant cells on histological examination [3, 4]. The GCF has an equal sex distribution, tends to occur among 20+-year olds, and occurs most often in Caucasians. Houston presented further validation for this lesion in 1982 [5]. A number of authors have disputed the need for the classification of the GCF as a separate entity from the fibroma. They based their conclusions on the fact that stellate and multinucleated cells are found at various stages of maturation of the lesion and that other histological features are not sufficiently unusual or characteristic to warrant identification as a distinct entity [6–9]. Most current pathology literature identifies these two lesions as distinct entities [10]. The purpose of this paper is to present a case in which both a giant cell fibroma and focal fibrous hyperplasia presented in the same patient at the same time.

## 2. Case Report

**Figure 1:**
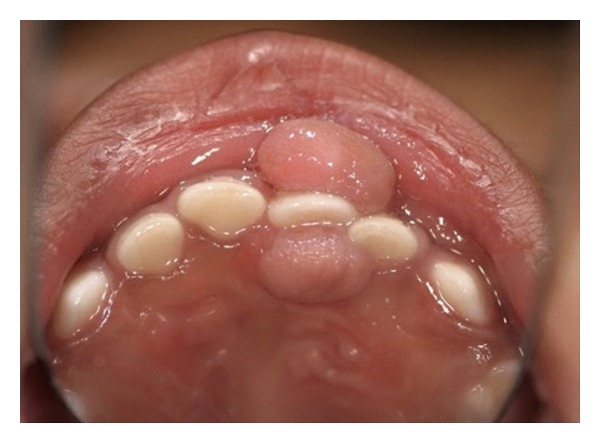
Buccal and palatal lesions.

A 17-month-old African-American girl was referred from her private dentist for evaluation of two papillomatous lesions in the anterior maxilla (Figure 1). History revealed a healthy child, the product of a normal, uncomplicated full-term pregnancy. Dental history revealed that the growths first appeared 5 months ago and were slowly increasing in size. No history of dental and/or facial trauma was reported. The child was not in any pain, and no interference with feeding was reported by the mother.

The lesion on the buccal mucosa was about 1 centimeter in size, pink in color, stippled and attached via a peduncle to the attached gingiva opposite tooth no. 51 and tooth no. 52. The lesion blanched slightly with digital pressure.

The lesion of the palate was about 0.75 cm in size, circular, pink in color, stippled, sessile, and adjacent to the incisive papilla between tooth no. 51 and tooth no. 52. No blanching was noted with slight digital pressure.

**Figure 2:**
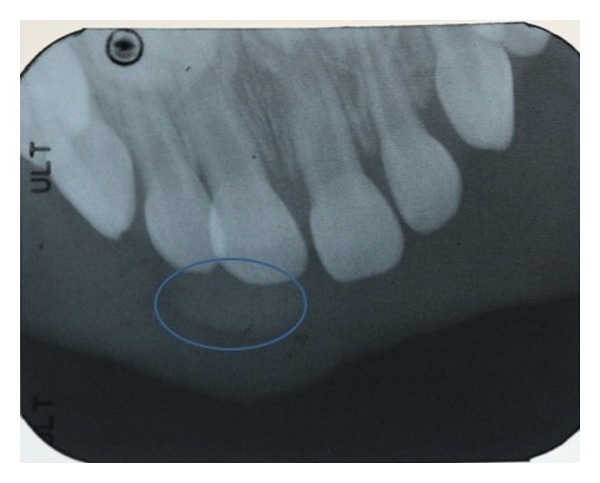
Radiographic image with palatal lesion circled.

A radiograph of the area revealed no bony involvement (Figure 2).

Due to the age of the patient and the relative complexity of the procedure, it was decided to perform excisional biopsy of both lesions under general anesthesia. The lesions were excised utilizing a number 11 scalpel blade. The excised areas were then cauterized. 

**Figure 3:**
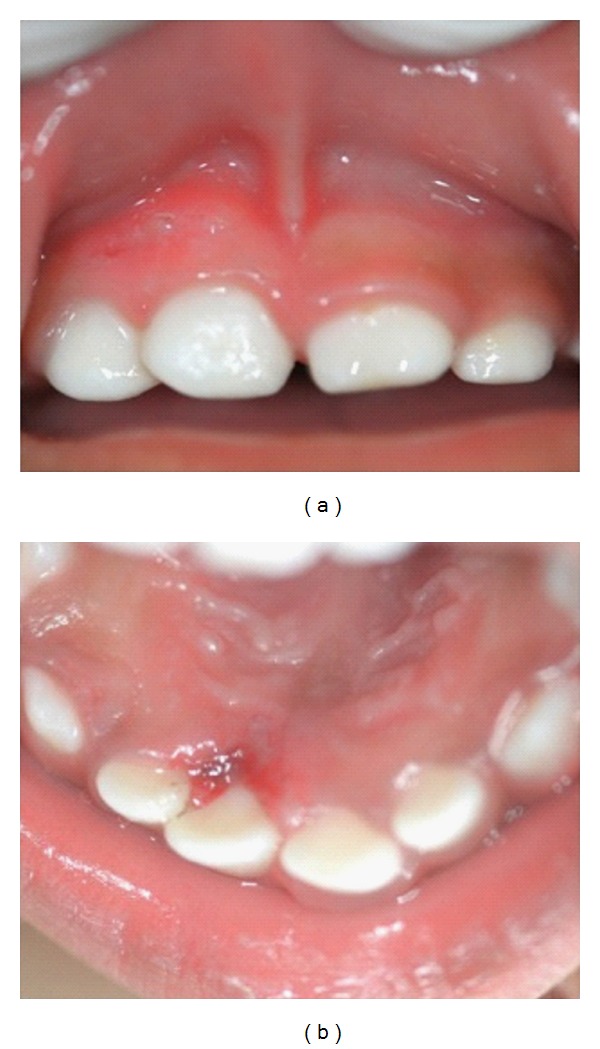
One-week postoperative pictures.

On one-week followup both areas were healing well; no pain or discomfort and no difficulty in eating were reported by the mother (Figure 3).

### 2.1. Differential Diagnosis

Table 1 includes most of the lesions that should be considered in the differential diagnosis of both lesions. An attempt is made to order the lesions ranging from the most likely to the least likely to occur in this specific patient.

### 2.2. Histology

**Figure 4:**
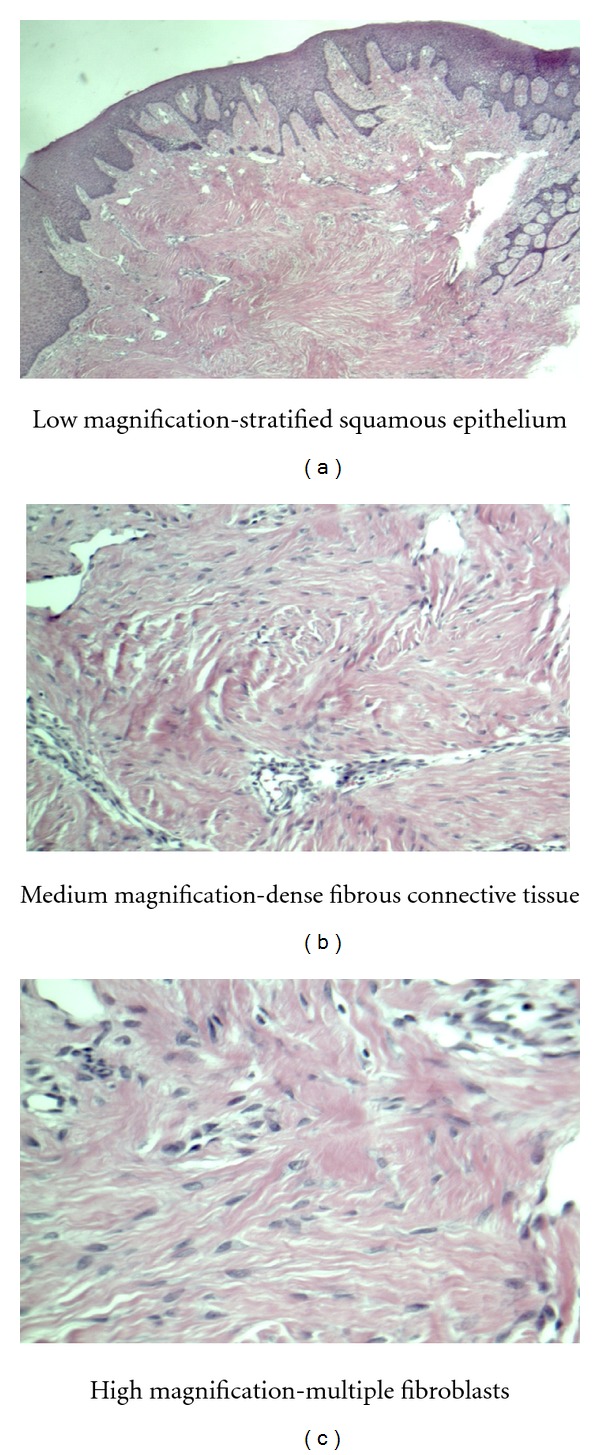
Histology slides of buccal lesion—focal fibrous hyperplasia (Fibroma).

Buccal lesion—dense fibrous connective tissue surfaced by stratified squamous epithelium with a normal maturation pattern. No evidence of malignancy. Diagnosis of focal fibrous hyperplasia (Figure 4).

**Figure 5:**
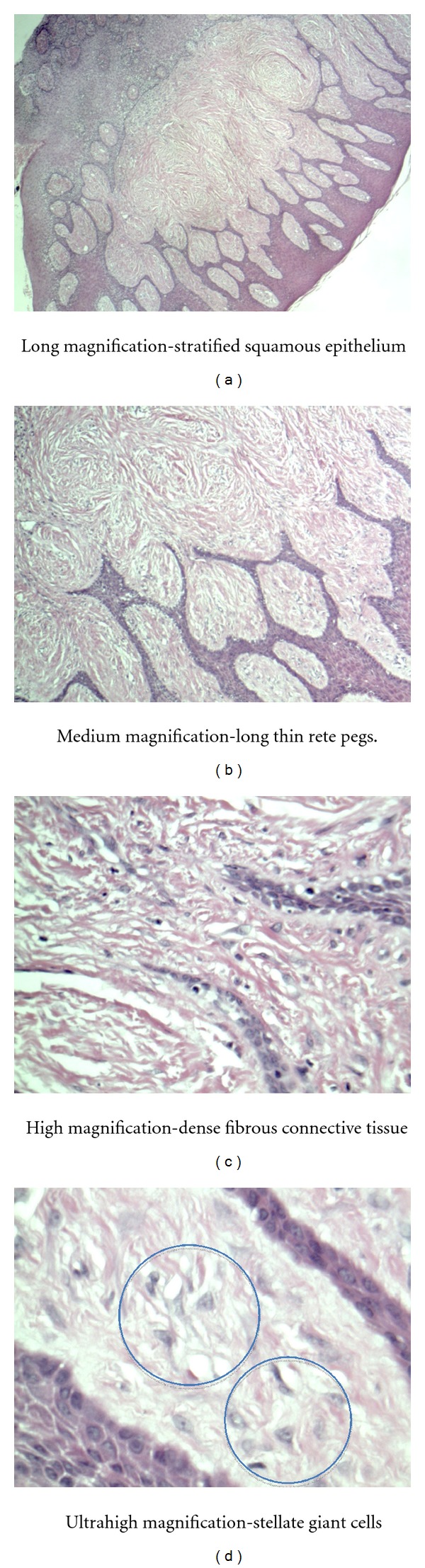
Histology slides of palatal lesion—giant cell fibroma.

Palatal lesion—dense fibrous connective tissue surfaced by stratified squamous epithelium with a normal maturation pattern. Many stellate fibroblasts and long thin rete pegs are present. Diagnosis of giant cell fibroma (Figure 5).

The pathology report also suggested that since there were only subtle histological differences between the two lesions, they might have simply been in different stages of maturation. The report also advised that the child be assessed now and in the future for additional lesions and to consider and rule out fibromatosis syndrome.

## 3. Discussion

As the most common nonneoplastic growth in the oral cavity, much has been written about the fibroma (FFH). The identification of a GCF as a separate entity by Weathers and Callihan added a new dimension to the discussion [3, 4]. The clinical presentation and epidemiology of most nonneoplastic growths in the oral cavity are quite similar; thus identification is dependant on histopathological differentiation. In this case report, two clinically distinct lesions presented in the same patient. As an African-American infant, this patient was outside the normal epidemiological predictors for the presentation of either lesion. The presentation of both types of lesion in the same patient has not been reported previously in the literature. The mother reported a 5-month period of observation of especially the buccal lesion. The similar size of the two lesions and the young age of the patient suggest that lifetime of the two lesions was similar. This would make a case for the Weathers and Callihan postulate that GCF is a separate entity and not merely a different presentation based on maturation of the lesion. Regezi et al. found that the presence of stellate cells is dependant on the pattern of collagen in the lamina propria and that stellate cells are most often found in oral lesions presenting on the gingiva or palate where the submucosa consists mainly of lamina propria [8]. Given the preponderance of lamina propria in the locations of these lesions, they should both have presented with stellate cells, according to Regezi et al. The fact that only one of the lesions presented with stellate cells gives further credence to the Weathers and Callihan postulate that GCF is a separate entity. The detractors for the identification of GCF as a separate entity also note that the proposed treatment, possible causative factors, and recurrence rate for both lesions are identical. On a histopathological level, GCF and FFH are distinct; on a clinical level the difference is insignificant. It is much more important to distinguish FFH/GCF from other nonneoplastic lesions that could have impact on developing structures or bone. This case report illustrates that clinical impact of these two lesions is similar and that on a patient care level making the histopathological distinction did not alter treatment.

## 4. Conclusions

Multiple fibrotic lesions can concurrently occur in young children.

GCF as a separate entity from FFH can be established histologically.

Treatment of the FFH and GCF is identical. 

## Figures and Tables

**Table 1:** Differential diagnosis.

Lesion	Age	Sex	Site	Surface	Presentation	Radiograph	Incidence	Size
Fibroma [10–13]	20+	F	Gingiva/buccal mucosa	Smooth Keratinized	Pedunculated or sessile	None	Common	1+ cm
Pyogenic granuloma [10–13]	20+	F	Gingiva	Ulcerated	Pedunculated	None	Common	2-3 cm
Papilloma [10, 13]	30+	M/F	Lips, tongue	Papillary	Pedunculated	None	Uncommon	Small
Peripheral ossifying fibroma [10–13]	10+	F	Interdental papilla	Smooth Keratinized	Pedunculated or sessile	None	Rare	>1 cm
Giant cell fibroma [3, 5, 7, 13, 14]	20+	M/F	Mandibular gingiva	Papillary	Pedunculated or sessile	None	Rare	>1 cm
Peripheral odontogenic fibroma [10, 13]	Any	M/F	Attached gingiva	Smooth	Pedunculated or sessile	Sometimes	Uncommon	1-2 cm
Peripheral adenomatoid odontogenic tumor [10, 13]	10+	F	Anterior maxilla	Smooth Keratinized	Nodular swelling	None	Rare	0.5–1 cm
Peripheral giant cell granuloma [10, 12, 13, 15]	<30	F	Gingiva/alveolar ridge	Ulcerated	Pedunculated or sessile	None	Rare	0.5–1 cm
Neurofibroma [10, 13]	45+	M/F	Gingiva or tongue	Smooth	Pedunculated or sessile	None	Rare	1–3 cm
Lipoma [10, 13]	40+	M	Parotid area or buccal mucosa	Smooth Keratinized	Sessile	None	Uncommon	0.5–3 cm
Peripheral ameloblastoma [10, 13]	50+	M	Posterior gingival	Smooth or pebbly	Sessile	Sometimes	Very rare	0.5–1 cm
Intraoral neurilemoma [10, 13]	Any	M/F	Tongue	Smooth Keratinized	Sessile	None	Uncommon	0.5–1 cm
Peripheral calcifying odontogenic cyst [10, 13]	60+	M	Anterior mandible	Smooth	Sessile	Erosion of bone	Very rare	0.5–1 cm
